# Comparative Physiology of the Brain and Comparative Psychology

**Published:** 1901-05

**Authors:** 


Comparative Physiology of the Brain and Comparative Psychology. By Jacques Loeb, M. D., Professor of Physiology in the University of Chicago. Duodecimo, pp. 309. Illustrated. New York: G. P. Putnam's Sons. 1900. [Price, $2.00.]

The author has written a work based upon actual experiments and close observation of the lower forms of animal life. The first chapter is devoted to spontaneity and coördination by many experiments on medusæ. This is followed by studies upon ascidians and actinians, echinoderms, worms, arthropods, mollusks and vertebrates.

These experiments are carefully reported and the reader is helped to understand them by numerous illustrations. We must confess, that to the average medical mind, much of the author's meaning must be obscure. This is due partly to the fact, that the average physician is not used to personally conducting physiological experiments, and also to the fact that we are not zoologists.

The second part of the book which deals with the theory of animal instincts, the relation of heredity to the central nervous system and memory, is extremely interesting and well presented.

His views are his own and he does not hesitate to announce them and defend them. He is not a Darwinian, when he asserts that pain sensations may appear in certain forms of life, without existing even in rudimentary form, in the whole animal kingdom. He combats the idea that consciousness exists in every animal and is present to a certain degree even in the egg. He also emphasises the statement that the idea of a steady, continuous development is inconsistent with the general physical qualities of protoplasm. "The colloidal substances in our protoplasm possess critical points."

By this he means that the colloids change their state very easily; that colloids lend themselves very readily to a discontinuous series of changes. This excludes the Darwinian theory of gradual, steady development. The author has presented his views in an attractive and forceful way, and we bespeak for him a wide reading.

